# Effects of phytase inclusion in diets containing rice protein concentrate (RPC) on the nutrient digestibility, growth and chemical characteristics of rohu (*Labeo rohita*)

**DOI:** 10.1371/journal.pone.0302859

**Published:** 2024-05-24

**Authors:** Ayesha Khizar, Mahroze Fatima, Noor Khan, Muhammad Afzal Rashid

**Affiliations:** 1 Department of Fisheries and Aquaculture, University of Veterinary and Animal Sciences, Lahore, Pakistan; 2 Institute of Zoology, University of Punjab, Lahore, Pakistan; 3 Department of Animal Nutrition, University of Veterinary and Animal Sciences, Lahore, Pakistan; University of Agriculture Faisalabad, PAKISTAN

## Abstract

The objective of the current study was to assess the impact of dietary phytase supplementation on *Labeo rohita* fingerlings and to examine the effects on growth, nutrient digestibility and chemical characteristics of diets containing rice protein concentrate (RPC) as a major protein source. Six experimental diets were made, i.e., a positive control (fishmeal-based diet with no phytase), FM_0_; a negative control (RPC-based diet with no phytase), RPC_0_; and four supplemental phytase levels (250, 500, 1000, and 2000 FTU/kg). Fingerlings with an average weight of 9.42 ± 0.02 grams (mean ± SD) were randomly distributed into six experimental groups of three replicates, each containing 25 fish per tank (75 liters of water), provided with experimental diets at a rate equivalent to 5% of their body weight for 90 days, and uneaten feed was collected after 2 hours to determine feed consumption. The feces were collected before feeding to estimate digestibility. Phytase in combination with the RPC-based diet significantly (p < 0.05) enhanced phytate phosphorus *in vitro* hydrolysis; growth performance; nutrient (crude protein, crude fat, moisture and gross energy) and mineral (P, Ca, Mg, Na, K, Zn, Mn and Cu) digestibility; digestive enzyme (protease, lipase and amylase) activity; and mineral deposition up to 1000 FTU/kg phytase. However, the hepatosomatic and viscerosomatic indices and carcass composition were not influenced (p > 0.05) by phytase supplementation. Increasing phytase supplementation in the RPC-based diets led to a significant (p < 0.05) decrease in the serum biochemical parameters (alkaline phosphatase activity, aspartate aminotransferase, alanine aminotransferase), which resulted in improved liver health. In conclusion, phytase-supplemented RPC-based diets improved the growth, mineral/nutrient digestibility, digestive enzymes, serum biochemistry, and mineral deposition of *L*. *rohita* fingerlings up to 1000 FTU/kg. Broken line regression analysis revealed that the optimum phytase concentration in the RPC-based diet for *L*. *rohita* was 874.19 FTU/kg.

## Introduction

Protein is a crucial component of aquafeeds, and fishmeal (FM) is widely acknowledged as the most suitable protein source due to its balanced amino acid profile [1]. However, the limited supply of FM and high cost have compelled aqua culturists to investigate alternative protein sources to fulfill the increasing demand for fish feed [2, 3]. Considering this situation, plant proteins (PPs) appear to be the most feasible choice, as they offer potential solutions to both cost and sustainability challenges in aquafeed production [4]. However, PP sources are associated with several problems, such as the presence of anti-nutritional factors (ANFs) [5].

Plant protein sources such as rice protein concentrate (RPC) are a byproduct of rice made from rice polish and contain ANF, specifically phytate. Some of the ANFs are denatured during the feed process; however, phytate is considered relatively heat stable and requires several enzymatic reactions for its breakdown [6, 7]. It forms a complex with proteins and affects the digestibility and intake of PP sources [6, 8]. RPC can be utilized as a protein source for fish feed. However, it contains remarkably high levels of phytate, which needs to be addressed [9].

To overcome this limitation, exogenous enzymes such as phytase have been proven to be effective in various PP meals [10–12]. Phytase supplementation in feed breaks down the phytate complex and releases bound phosphorus (P), leading to improved nutrient digestibility and availability from PP sources to fish. It also enhances P utilization, alleviates the excretion of P into the environment, and decreases water pollution [6, 7]. Moreover, it also enhances the absorption of chelated nutrients and minerals from the intestinal mucosa of fish [8, 13, 14]. Due to this potential, the utilization of phytase is currently being extensively assessed [15]. Various studies have been conducted on phytase supplementation in plant-based diets, which have shown enhanced growth performance and nutrient utilization in fish species [16–19]. However, no study on RPC-based diets has been reported.

*Labeo rohita* is one of the most widely cultured fish species in Asia due to its high nutritional value, delicious taste, low economic value, and high market value. Owing to its high demand, this species is cultured in intensive systems on formulated feeds. Therefore, this study aimed to evaluate the effects of phytase supplementation on the growth performance, digestibility, *in vitro* phytate hydrolysis, carcass composition, mineral content, serum biochemistry, and digestive enzyme activity of *L*. *rohita* fed RPC-based diets. Understanding the impact of phytase in this context can contribute to the development of more efficient and sustainable aquafeed formulations, fostering the growth of aquaculture to meet the demands of a growing global population.

## Materials and methods

### Ethics approval

Ethics approval (No. DR/677) for the experiment was obtained from the Ethical Review Committee of the University of Veterinary and Animal Sciences (UVAS), Lahore, Pakistan.

### Study area and experimental fish

The study took place at the Fish Seed Rearing Unit, Ravi Campus, University of Veterinary and Animal Sciences, Pattoki, and *L*. *rohita* fingerlings with an initial weight of 9.42±0.02 g were used as the experimental fish.

### Fish acclimatization

The fish fingerlings were procured from the ponds of the C block, Ravi campus, UVAS. To protect the fish from potential pathogens, the fingerlings were subjected to a bath solution containing 5 g/L KMnO_4_ to create an environment that would minimize the risk of infections and diseases. After this, the fingerlings underwent a fifteen-day acclimatization period under laboratory conditions. Throughout this period, the fingerlings were provided a basal (RPC_0_) diet containing 30% crude protein (CP) for feeding.

### Experimental diet preparation

Rice protein concentrate was purchased from Qurashi Brothers, Karachi, Pakistan, and its proximate composition was analyzed (Table 1) following the standard method of the AOAC [20]. The phytate content in the RPC ingredient and experimental diets were analyzed using a colorimetric method following Latta and Eskin [21]. In the feeding trial, the diets were made using RPC as the main ingredient, which contained 30% CP supplemented with different phytase (FTU/kg) concentrations, i.e., 0 (RPC_0_), 250 (RPC_250_), 500 (RPC_500_), 1000 (RPC_1000_), and 2000 (RPC_2000_). The FM-based diet was used as a positive control (FM_0_). For the preparation of the experimental diets, first, the dry feed ingredients (Table 1) were ground in an electric grinder (KENWOOD, AT284) to a fine powder, sieved (0.05 mm), and then mixed with the help of an electric mixer (KENWOOD, AT283). During the mixing process, the dry ingredients were supplemented with fish oil, a mineral mixture, vitamin premix, and chromic oxide (a marker for digestibility). Afterward, a dough was prepared by incorporating 15% water. The pellets were subsequently produced using a meat mincer (ANEX, AG 3060) and then shade-dried with up to 10% moisture [22]. Different dilutions of phytase (Microtech 10000 Plus granular, phytase 10,000 FTU/g, China, Pakistan) with distilled water (according to the experimental design) were sprayed on the pellets. Again, the pellets were dried and subsequently stored in airtight bags. The AOAC standard method [20] was used to determine the proximate composition of the RPC and experimental diets. The crude fat and CP contents were analyzed following the ether extraction method through a Soxhlet apparatus (behr Labor-Technik, Germany) and a Kjeldahl apparatus (FOSS Analytical A/S) after acid digestion, respectively. The ash and dry matter contents were determined on a muffle furnace (Eyela, TMF 3100) at 660°C and in a hot air oven at 105°C, respectively. Moreover, the gross energy content was quantified using an adiabatic oxygen bomb calorimeter (Parr Instrument Co., Moline. USA).

**Table 1:** Composition of experimental diets (%) and RPC ingredient of *Labeo rohita* fingerlings.

Ingredients*		Phytase levels in diets (FTU/kg)					
	RPC	FM_0_	RPC_0_	RPC_250_	RPC_500_	RPC_1000_	RPC_2000_
Fish meal		45.00	5.00	5.00	5.00	5.00	5.00
Rice protein concentrate		-	32.00	32.00	32.00	32.00	32.00
Sunflower meal		10.00	10.00	10.00	10.00	10.00	10.00
Corn gluten (60%)		4.50	8.50	8.50	8.50	8.50	8.50
Wheat flour		20.00	20.00	20.00	20.00	20.00	20.00
Rice bran		11.00	15.00	15.00	15.00	15.00	15.00
Fish oil^a^		7.00	7.00	7.00	7.00	7.00	7.00
Vitamin premix^b^		1.00	1.00	1.00	1.00	1.00	1.00
Mineral mixture^c^		1.00	1.00	1.00	1.00	1.00	1.00
Chromic oxide		0.50	0.50	0.50	0.50	0.50	0.50
Phytase (g/kg)^d^		-	-	25.00 (250.0 FTU/kg)	50.00 (500.0 FTU/kg)	100.00 (1000.0 FTU/kg)	200.00 (2000.0 FTU/kg)
**Proximate Composition (on dry basis, %)**							
Dry matter	93.00	90.30	90.22	90.41	90.31	90.22	90.25
Crude protein	73.60	30.12	30.03	30.02	30.14	30.07	30.09
Crude fat	10.20	10.89	10.81	10.82	10.81	10.84	10.85
Ash	3.90	4.34	3.93	3.94	4.23	4.32	4.23
Gross Energy (Kcal/kg)	5318.00	4660.00	4740.00	4740.00	4740.00	4740.00	4740.00

^a^Fish oil = cod liver oil (poultry-vet Co, Nazimabad, Karachi, Pakistan).

^b^Each kg of vitamin premix contains: Vitamin A 15 M.I.U, Vitamin D3 3 M.I.U, Nicotinic acid 25000 mg, Vitamin B1 5000 mg, Vitamin E 6000IU, Vitamin B2 6000 mg, Vitamin K3 4000 mg, Vitamin B6 4000 mg, Folic acid 750 mg, Vitamin B12 9000 mg, Vitamin C 15000 mg, Calcium Pentothenate 10000 mg

^c^Each kg of a Mineral mixture contains: KH_2_PO_4_ 479 mg/g, MgSO_4_.7H_2_O 153 mg/g, CoCl.6H_2_O 0.0816 mg/g, NaCl 51 mg/g, AlCl_3._6H_2_O 0.255 mg/g CuSo_4_ .5H_2_O 210.67 mg/g, FeSo_4_.H_2_O 100.67 mg/g, MnSo_4._5H_2_O 116.67 mg/g, ZnSO_4._7 H_2_O 121.33 mg/g CaCO_3_ 316 mg/g and Cellulose 65 mg/g

^d^Phytase = Microtech 10000 Plus granular, phytase 10,000 FTU/g, China, Pakistan.

*The rice protein concentrate was purchased from Qurashi brothers, Lahore, Pakistan. While other ingredients were purchased from Gazi Brothers (Pvt. Ltd), Lahore, Pakistan.

The phytate content was found to be 8.72 g/kg (RPC ingredient), 3.3 g/kg (FM-based diet), and 6.8 g/kg (RPC-based diets).

Abbreviations: FM = Fishmeal; RPC = Rice protein concentrate

### Rearing and feeding conditions

This experiment was conducted in a V-shaped steel tank placed indoors, and a natural photoperiod (8 h) was maintained throughout the experimental period. After acclimatization, the total fish fingerlings (n = 450) were stocked randomly into 18 V-shaped steel tanks (25 fish/tank) with 75 liters of water. The fish were fed experimental diets at 5% body weight for 90 days, and uneaten feed (feed waste) was collected after 2 hours to determine feed intake. For digestibility measurements, the sedimented feces were collected daily before feeding the fish by opening the valve at the bottom of each tank. Continuous aeration was given for the maintenance of dissolved oxygen (DO) within the optimum range (5.8–7.3 mg/L). The other water quality parameters, including temperature and pH, were monitored (Model 55, YSI, Inc., Yellow Springs, Ohio, USA), and average values were observed at 24.9–28.7°C and 7.4–8.6, respectively, throughout the experimental trial.

### Sample collection

After the 90-day trial, the fish were subjected to a 24-hour fasting period, and the final weight of the fish in each tank was recorded for growth performance analysis. Subsequently, they were anesthetized using 150 mg/L tricane methanesulfate (MS 222, Sigma‒Aldrich) following the methods of Khan et al. [23]. Three fish from each tank were collected, pooled and placed in a hot air oven for carcass analysis, and another 3 fish were used for whole-body mineral analysis. For serum biochemical analysis, 5 fish were used, and blood was collected from the caudal vein in a 3 mL plain tube (BD vacutainers) and centrifuged for 15 minutes at 18,000 × g at 4°C. After centrifugation, the serum was collected in Eppendorf tubes for further analysis. Five additional fish were dissected, and their liver and viscera were measured for biological indices. The remaining 7 fish were dissected, and their intestine and bone samples were separated. The collected intestines were washed with distilled water and stored in sucrose solution at -20°C for further analysis of digestive enzyme activity. The bone samples were also stored at -20°C until analysis.

### Growth performance and body indices

The growth performance was calculated in terms of average weight gain (AWG), weight gain (%), feed conversion ratio (FCR), specific growth rate (SGR), survival rate (SR), feed intake, and protein efficiency ratio (PER) using the following formulas:

AWG(g)=averagefinalbodyweight(g)−averageinitialbodyweight(g)

$$Weight\;gain\;\%=\frac{final\;body\;weight-initial\;body\;weight\;(g)}{initial\;body\;weight}\times 100$$

$$FCR=\frac{total\;dry\;feed\;intake\;(g)}{wet\;weight\;gain\;(g)}$$

$$SGR\;(\frac{\%}{day})=\frac{\ln\left(final\;body\;weight)-\ln(initial\;body\;weight\right)}{no.of\;days}\times 100$$

$$SR(\%)=\frac{final\;fish\;number}{initial\;fish\;number}\times 100$$

Feedintake(g)=feedgiven(g)−unconsumedfeed(g)

$$PER=\frac{AWG\;(g)}{Protein\;intake\;(g)}$$

The viscerosomatic index (VSI) and hepatosomatic index (HSI) were determined from the liver and viscera weights using the following formulas.


$$VSI\;(\%)=(\frac{Viscera\;weight\;(g)}{Whole\;body\;weight\;(g)})\times 100$$

$$HSI\;(\%)=\left(\frac{Liver\;weight\;(g)}{Whole\;body\;weight\;(g)}\right)\times \;100$$

### Carcass composition

The fish carcass composition (CP, crude fat, ash, and moisture) was determined following the standard method of the AOAC [20] as described in the experimental diet preparation section.

### Mineral analysis

The dried whole-body and stored bone samples were subjected to mineral analysis. The collected bone samples were further processed, their soft tissues and muscles were separated, and only bones and spines were left after 2–3 min of boiling. After boiling, the collected bone samples were cleaned, rinsed, and dried in an oven and then subjected to ether extraction to eliminate fat. Finally, the bone samples from each replicate were dried, ground, and pooled together for mineral analysis. The samples (whole body and bones) were further subjected to wet digestion using a mixture of nitric acid and perchloric acid at a ratio of 3:1 following the AOAC [20] standard method, and the mineral [calcium (Ca), zinc (Zn), copper (Cu), magnesium (Mg), and manganese (Mn)] contents were determined through atomic absorption spectrophotometry (Hitachi Polarized Zeeman AAS, Z-8200, Japan); moreover, the P content in the samples was quantified using a UV visible spectrophotometer (U-2001, Hitachi), while the sodium (Na) and potassium (K) contents were determined via a flame photometer (Jenway, PFP 7, UK).

### Nutrient digestibility and mineral absorption

The pooled fecal matter from each replicate and their respective test diets were dried in an oven at 60°C, ground, and pooled for analysis of nutrients and mineral digestibility. The apparent digestibility coefficient (ADC) of nutrients and mineral absorption was subsequently determined by the following formula.


$$ADC\;(\%)=100-100\;x\;\frac{\left(Percent\;marker\;in\;diet\;x\;Percent\;nutrient\;in\;feces\right)}{\left(Percent\;marker\;in\;feces\;x\;Percent\;nutrient\;in\;diet\right)}$$

### Serum biochemical parameters

Serum biochemical parameters, such as alkaline phosphatase (ALP) (Cat no. BS:1/AP05.020.0100), aspartate aminotransferase (AST) (Cat no. BS:1/OT04.025.0100), and alanine aminotransferase (ALT) (Cat no. BS:1/PT04.0100) activity, were analyzed using specific kits from ARENA BioScien, Egypt, following Xu et al. [24].

### Intestinal digestive enzyme analysis

The intestine samples from 7 fish were collected, pooled, and homogenized in a sucrose solution, after which the enzyme extract was separated via centrifugation for digestive enzyme analysis. The activity of amylase was measured by using a starch solution as a substrate at 2% (w/v) [25]. A spectrophotometric method was used to measure the activity of lipase using the substrate p-nitro phenyl palmitate (pNPP) [26]. The protease activity was calculated using Kunitz’s [27] method for casein digestion. The crude enzyme extract hydrolyzes casein and produces a color equivalent to that of 1 μmol/min tyrosine (pH = 7.5) at 37°C. The soluble protein concentration was measured in diluted homogenates following the Bradford method [28] using bovine serum albumin (BSA) as the standard. The enzymatic activity was measured as U/mg protein.

### *In vitro* phytate hydrolysis

*In vitro* phytate hydrolysis was measured following the methods of Baruah et al. [29]. A total of 0.5 g of RPC was mixed with an adequate amount of water, and then different dilutions of phytase were added (0, 250, 500, 1000, and 2000 FTU/kg). These mixtures were incubated at 37°C for 1 hour in triplicate. After incubation, the phytate P content of the samples was further determined [29]. Briefly, the P content was determined using the wet digestion method, and the P content was analyzed using a spectrophotometer.

### Statistical analysis

The experiment was designed as a completely randomized design (CR Design), and the results were analyzed using one-way ANOVA. The linearity of the data was checked using the Levene test (homogeneity) and the Shapiro‒Wilk test (normality). Moreover, the different phytase levels were taken as fixed factors, while the allocation of fish to the treatment groups (tanks) was randomized (random factor). The mean values were compared using Tukey’s honestly significant difference (HSD) test if significant differences among the treatments were observed. Broken line regression analysis was used to evaluate the optimum requirement of phytase in the fish diet [30]. CoStat computer software (version 6.303) was used for one-way analysis, while broken-line regression analysis was performed in R Studio (version 2023.03.0). The results were considered significant at p<0.05.

## Results

### *In vitro* phytate hydrolysis

The *in vitro* hydrolysis of phytate P increased with increasing levels of phytase up to 1000 FTU/kg in the RPC diets (Table 2).

**Table 2:** Effect of phytase supplementation on phytate phosphorus hydrolysis of rice protein concentrate.

Parameters	Phytase levels in diets (FTU/kg)					PSE	p value
	RPC_0_	RPC_250_	RPC_500_	RPC_1000_	RPC_2000_		
Phytate phosphorus (mg/kg)	2.37^c^	2.45^c^	2.56^b^	2.70^a^	2.68^a^	0.02	<0.01

The upper superscripts showed statistically significant differences at p<0.05 while the no superscript showed nonsignificant results (p>0.05)

PSE = Pooled standard error = √MSE/n (where MSE = mean‐squared error)

Abbreviations: RPC = Rice protein concentrate

### Growth performance and body indices

**Fig 1:**
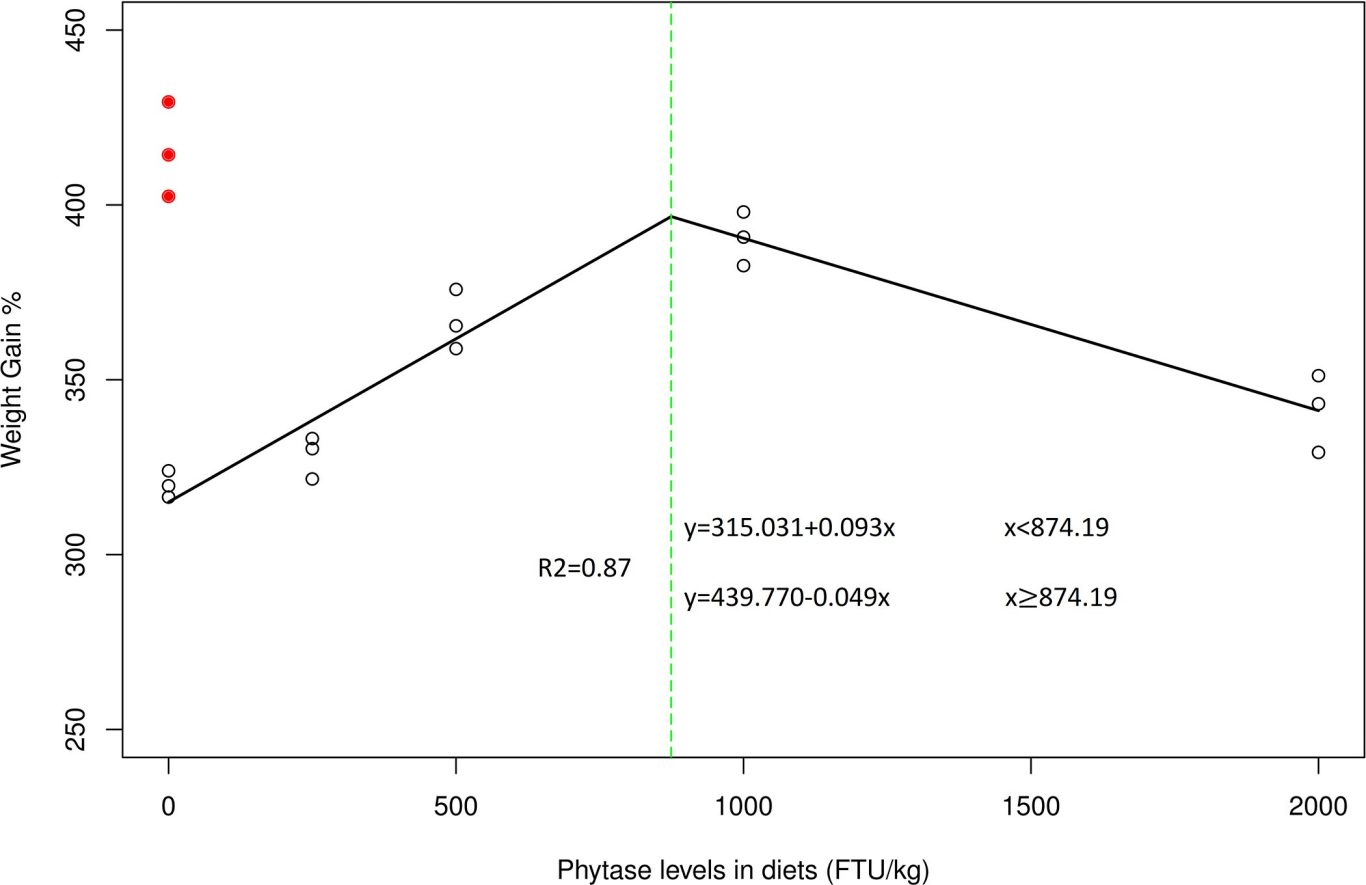
The optimum level of phytase supplementation for *Labeo rohita* fed a rice protein concentrate (RPC)-based diet was determined via broken-line regression analysis of weight gain (%) data. The red dotted points indicate diets comprising FM_0_ (a fishmeal-based diet with no phytase supplementation), while others showed different levels of phytase in the RPC-based diet.

The highest WG% (390.47%), SGR (1.76%) and PER (3.02) and lowest FCR (1.10) were observed at the 1000 FTU/kg phytase level, which was not significantly different from those of the 500 FTU/kg diet (WG% (366.73%), SGR (1.71%), PER (2.81)) and FCR (1.18) compared to those of the other phytase-supplemented RPC-based diets. However, no significant effects of phytase supplementation were observed on the FI or SR (Table 3). Moreover, the optimum dietary level of phytase was calculated to be 874.19 FTU/kg according to the weight gain (%) data obtained via broken-line regression analysis (R^2^ = 0.87) (Fig 1).

**Table 3:** Growth performance and body indices of *Labeo rohita* fingerlings fed rice protein concentrate based diets.

Parameters	Phytase levels in diets (FTU/kg)						PSE	p value
	FM_0_ (positive control)	RPC_0_	RPC_250_	RPC_500_	RPC_1000_	RPC_2000_		
IW (g)	9.42	9.42	9.44	9.42	9.44	9.45	0.01	0.0864
FW (g)	48.55^a^	39.55^d^	40.45^d^	43.96^bc^	46.33^ab^	41.67^cd^	0.52	<0.01
AWG (g)	39.13^a^	30.14^d^	31.01^d^	34.55^bc^	36.88^ab^	32.23^cd^	0.51	<0.01
WG (%)	415.41^a^	320.03 3.76^c^	328.37^c^	366.73^b^	390.47^b^	341.17^c^	5.21	<0.01
SGR (%/day)	1.82^a^	1.59^c^	1.61^c^	1.71^b^	1.76^ab^	1.64^c^	0.01	<0.01
FCR (g/g)	1.04^d^	1.35^a^	1.31^a^	1.18^bc^	1.10^cd^	1.26^ab^	0.02	<0.01
FI (g)	40.67	40.76	40.60	40.96	40.67	40.90	0.40	0.9836
PER	3.21^a^	2.46^d^	2.54^cd^	2.81^bc^	3.02^ab^	2.62^cd^	0.06	<0.01
Survival rate (%)	100	97.33	100	100	100	98.67	1.22	0.5464
**Body Indices (%)**								
HSI	2.95	2.82	2.83	2.88	2.91	2.89	0.22	0.9979
VSI	7.34	6.66	6.69	6.83	7.29	7.26	0.31	0.4471

The superscripts showed statistically significant differences at p<0.05 while the cells within the same row containing shared superscript letters have no statistically significant difference (p > 0.05).

PSE = Pooled standard error = √MSE/n (where MSE = mean‐squared error)

Abbreviations: FM = Fish meal; RPC = Rice protein concentrate; IW = Initial weight; FW = Final weight; AWG = Average weight gain; WG% = weight gain %; SGR = Specific growth rate; FCR = Feed conversion ratio; FI = Feed intake; PER = Protein efficiency ratio; HSI = Hepatosomatic index; VSI = Viscerosomatic index

The body indices (HSI and VSI) did not significantly differ among the groups supplemented with phytase and those fed the RPC-based diet (Table 3).

### Carcass composition

The addition of phytase to the RPC-based diets did not significantly impact the carcass composition in terms of moisture, CP, CF, or ash content of *L*. *rohita*. Moreover, the average carcass compositions of the RPC-based diets were determined to be moisture (74.60%), CP (16.80%), CF (2.67%), and ash (4.79%) (Table 4).

**Table 4:** Effect of phytase supplementation on the fish carcass composition (%) of *Labeo rohita* fingerlings fed rice protein concentrate based diets.

Parameters(on wet base (%))	Phytase levels in diets (FTU/kg)						PSE	p value
	FM_0_	RPC_0_	RPC_250_	RPC_500_	RPC_1000_	RPC_2000_		
Moisture	74.78	74.80	74.65	74.31	74.79	74.46	0.16	0.241
CP	17.11	16.59	16.71	16.93	17.09	16.70	0.19	0.310
CF	3.00	2.62	2.58	2.74	2.84	2.61	0.11	0.112
Ash	5.00	4.68	4.66	5.03	4.99	4.62	0.11	0.609

Means showed significant differences at p < 0.05 while; without superscripts showed no significant difference among the treatments (p > 0.05)

PSE = Pooled standard error = √MSE/n (where MSE = mean‐squared error)

Abbreviations: FM = Fish meal; RPC = Rice protein concentrate; CP = Crude protein; CF = Crude fat

### Mineral composition (whole body and bone)

The fish fed diets containing 1000 FTU/kg and 2000 FTU/kg phytase exhibited significant improvements in whole-body mineral content, such as Ca (14.82 mg g^-1^), P (14.85 mg g^-1^), Mg (1.83 mg g^-1^), Na (4.41 mg g^-1^), and K (8.19 mg g^-1^). Zn (83.50 μg g^-1^), Cu (7.49 μg g^-1^) and Mn (37.60 μg g^-1^) significantly improved in the 1000 FTU/kg treatment group compared to the control group; these included Ca (13.26 mg g^-1^), P (12.12 mg g^-1^), Mg (0.47 mg g^-1^), Na (3.02 mg g^-1^), K (6.73 mg g^-1^), Zn (74.30 μg g^-1^), Cu (5.26 μg g^-1^) and Mn (24.96 μg g^-1^). Furthermore, the highest bone mineral contents were also detected in the 1000FTU/kg phytase supplemented diet for Ca (89.39 mg g^-1^), P (64.81 mg g^-1^), Mg (1.87 mg g^-1^), Na (7.60 mg g^-1^), K (9.80 mg g^-1^), Zn (152.15 μg g^-1^), Cu (8.38 μg g^-1^) and Mn (47.94 μg g^-1^) compared to those in the control group, such as Ca (73.85 mg g^-1^), P (47.12 mg g^-1^), Mg (1.54 mg g^-1^), Na (3.86 mg g^-1^), K (6.95 mg g^-1^), Zn (139.11 μg g^-1^), Cu (4.02 μg g^-1^) and Mn (38.75 μg g^-1^) (Table 5).

**Table 5:** Effect of phytase supplementation on the fish whole body and bones minerals composition of *Labeo rohita* fingerlings fed rice protein concentrate based diets.

Parameters	Phytase levels in diets (FTU/kg)						PSE	p value
	FM_0_	RPC_0_	RPC_250_	RPC_500_	RPC_1000_	RPC_2000_		
**Whole body**								
Ca (mg g^-1^)	14.85^a^	13.26^d^	13.44^c^	13.96^b^	14.82^a^	14.84^a^	0.02	<0.01
P (mg g^-1^)	14.88^a^	12.12^d^	13.28^c^	13.73^b^	14.85^a^	14.82^a^	0.02	<0.01
Mg (mg g^-1^)	1.88^a^	0.47^e^	0.88^d^	1.25^c^	1.83^ab^	1.78^b^	0.02	<0.01
Na (mg g^-1^)	4.48^a^	3.02^d^	3.53^c^	3.75^b^	4.41^a^	4.39^a^	0.02	<0.01
K (mg g^-1^)	8.23^a^	6.73^d^	7.29^c^	7.64^b^	8.19^a^	8.13^a^	0.02	<0.01
Zn (ug g^-1^)	83.57^a^	74.30^e^	79.93^d^	81.26^c^	83.50^a^	82.18^b^	0.03	<0.01
Cu (ug g^-1^)	7.57^a^	5.26^e^	5.94^d^	6.23^c^	7.49^a^	6.70^b^	0.03	<0.01
Mn (ug g^-1^)	37.65^a^	24.96^e^	29.95^d^	35.77^c^	37.60^a^	34.86^b^	0.02	<0.01
**Bones**								
Ca (mg g^-1^)	89.45^a^	73.85^e^	79.95^d^	83.17^c^	89.39^a^	86.87^b^	0.02	<0.01
P (mg g^-1^)	64.88^a^	47.12^e^	55.54^d^	59.19 ^c^	64.81^ab^	64.75^b^	0.02	<0.01
Mg (mg g^-1^)	1.91^a^	1.54^d^	1.63^c^	1.69^c^	1.87^ab^	1.81^b^	0.02	<0.01
Na (mg g^-1^)	7.65^a^	3.86^e^	4.44^d^	5.75 ^c^	7.60^a^	6.85^b^	0.02	<0.01
K (mg g^-1^)	9.86^a^	6.95^d^	8.77^c^	8.85^c^	9.80^ab^	9.76^b^	0.02	<0.01
Zn (ug g^-1^)	152.23^a^	139.11^e^	145.48^d^	148.52^c^	152.15^a^	150.25^b^	0.02	<0.01
Cu (ug g^-1^)	8.46^a^	4.02^e^	4.63^d^	5.87^c^	8.38^a^	6.85^b^	0.02	<0.01
Mn (ug g^-1^)	48.01^a^	38.75^e^	40.41^d^	42.69^c^	47.94^a^	44.91^b^	0.02	<0.01

The superscript letters showed statistically significant differences at p < 0.05, while the cells within the same row containing shared superscript have no statistically significant difference (p > 0.05).

PSE = Pooled standard error = √MSE/n (where MSE = mean‐squared error)

Abbreviations: FM = Fish meal; RPC = Rice protein concentrate; Ca = Calcium; P = Phosphorus; Mg = Magnesium; Na = Sodium; K = Potassium; Zn = Zinc; Cu = Copper; Mn = Manganese

### Nutrient digestibility and mineral absorption

The supplemental level of phytase at 1000 FTU/kg resulted in the maximum values of ADC in terms of dry matter, CP, CF, and gross energy in *L*. *rohita* fingerlings fed RPC-based diets, and these values decreased thereafter (Table 6). However, the mineral absorption of Ca (64.83–68.79%), P (64.35–70.39%), Na (36.76–45.61%), Mg (49.66–69.55%), and K (62.98–73.69%) increased up to 1000 FTU/kg. Moreover, the Zn (40.68–60.24%), Cu (44.86–64.46%), and Mn (41.89–63.49%) contents increased with increasing supplemental phytase (2000 FTU/kg) in the RPC-based diet (p<0.01) (Table 7).

**Table 6:** Effect of phytase supplementation on the apparent nutrient digestibility coefficient (ADC %) of *Labeo rohita* fingerlings fed rice protein concentrate based diets.

Parameters	Phytase levels in diets (FTU/kg)						PSE	p value
	FM_0_	RPC_0_	RPC_250_	RPC_500_	RPC_1000_	RPC_2000_		
DM	80.86^a^	71.70^e^	74.79^d^	76.61^c^	79.17^b^	77.71^c^	0.27	<0.01
CP	90.87^a^	79.16^d^	83.48^c^	87.70^b^	90.54^a^	87.43^b^	0.17	<0.01
CF	91.78^a^	77.32^d^	81.95^c^	86.98^b^	90.68^a^	88.15^b^	0.37	<0.01
GE	82.58^a^	70.05^f^	73.32^e^	76.52^d^	80.21^b^	78.21^c^	0.30	<0.01

The superscript letter in the rows showed significantly different results (p < 0.05), while the shared superscripts showed nonsignificant results (p > 0.05) in the diets.

PSE = Pooled standard error = √MSE/n (where MSE = mean‐squared error)

Abbreviations: FM = Fish meal; RPC = Rice protein concentrate; DM = Dry matter; CP = Crude protein; CF = Crude fat; GE = Gross energy

**Table 7:** Effect of phytase supplementation on mineral absorption(%) of *Labeo rohita* fingerlings fed rice protein concentrate based diets.

Parameters	Phytase levels in diets (FTU/kg)						PSE	p value
	FM_0_	RPC_0_	RPC_250_	RPC_500_	RPC_1000_	RPC_2000_		
Ca	68.88^a^	64.83^d^	66.44^c^	66.54^c^	68.79^ab^	68.72^b^	0.03	<0.01
P	70.47^a^	64.35^e^	66.75^d^	68.36^c^	70.39^ab^	70.33^b^	0.02	<0.01
Mg	69.62^a^	49.66^e^	51.85^d^	62.74^c^	69.55^ab^	69.46^b^	0.02	<0.01
Na	45.65^a^	36.76^e^	38.46^d^	41.64^c^	45.61^ab^	45.56^b^	0.02	<0.01
K	73.74^a^	62.98^e^	66.63^d^	69.75^c^	73.69^ab^	73.62^b^	0.02	<0.01
Zn	60.28^a^	40.68^d^	43.54^c^	45.83^b^	60.21^a^	60.24^a^	0.02	<0.01
Cu	64.46 ^a^	44.86^d^	48.94^c^	51.86^b^	64.44^a^	64.46^a^	0.01	<0.01
Mn	63.55^a^	41.89^d^	45.62^c^	49.70^b^	63.47^a^	63.49^a^	0.02	<0.01

The superscripts showed statistically significant differences at p < 0.05, while the shared superscripts within row showed no statistically significant difference (p > 0.05) in the diets.

PSE = Pooled standard error = √MSE/n (where MSE = mean‐squared error)

Abbreviations: FM = Fish meal; RPC = Rice protein concentrate; Ca = Calcium; P = Phosphorus; Mg = Magnesium; Na = Sodium; K = Potassium; Zn = Zinc; Cu = Copper; Mn = Maganese

### Serum biochemical parameters

The serum biochemical parameters (ALP, ALT, and AST activities) were significantly lower in *L*. *rohita* after up to 1000 FTU/kg phytase supplementation in RPC-based diets. However, there was no significant difference between the groups fed diets supplemented with phytase above 1000 FTU/kg and those fed the other RPC-based diets (Table 8).

**Table 8:** Effect of phytase supplementation on serum biochemistry of *Labeo rohita* fed rice protein concentrate based diet.

Parameters	Phytase levels in diets (FTU/kg)						PSE	p value
	FM_0_	RPC_0_	RPC_250_	RPC_500_	RPC_1000_	RPC_2000_		
ALP (U/L)	85.30^d^	90.86^a^	89.28^b^	87.65^c^	86.25^d^	85.46^d^	0.29	<0.01
ALT (U/L)	25.59^c^	28.52^a^	27.86^ab^	27.27^b^	25.50^c^	26.08^c^	0.19	<0.01
AST (U/L)	12.58^c^	15.75^a^	14.02^b^	13.98^b^	12.55^c^	12.68^c^	0.18	<0.01

The superscripts showed statistically significant differences at p < 0.05. while cells within the same row containing shared subscript have no statistically significant difference (p > 0.05)

PSE = Pooled standard error = √MSE/n (where MSE = mean‐squared error)

Abbreviations: FM = Fish meal; RPC = Rice protein concentrate; ALP = Alkaline phosphate activity; ALT = Alanine aminotransferase activity; AST = Aspartate aminotransferase activity

### Intestinal digestive enzyme analysis

The enzyme activities, i.e., protease, lipase, and amylase activities, were significantly greater in the 1000 FTU/kg and 2000 FTU/kg phytase supplementation groups than in the lower level group (Table 9).

**Table 9:** Effect of phytase supplementation on the digestive enzyme activity of *Labeo rohita* fingerlings fed rice protein concentrate based diet.

Parameters	Phytase levels in diets (FTU/kg)						PSE	p value
	FM_0_	RPC_0_	RPC_250_	RPC_500_	RPC_1000_	RPC_2000_		
Protease (U/mg protein)	0.96^a^	0.56^e^	0.66^d^	0.76^c^	0.87^b^	0.82^bc^	0.01	<0.01
Lipase (U/mg protein)	0.98^a^	0.44^d^	0.56^c^	0.69^b^	0.97^a^	0.94^a^	0.01	<0.01
Amylase (U/mg protein)	2.74^a^	2.07^d^	2.14^c^	2.32^b^	2.70^a^	2.69^a^	0.01	<0.01

Statistical significance, denoted by the superscripts was observed at p < 0.05, indicating notable differences. While the cells within the same row containing shared superscript letters have no statistically significant difference (p > 0.05) among the treatments

PSE = Pooled standard error = √MSE/n (where MSE = mean‐squared error)

Abbreviations: RPC = Rice protein concentrate; FM = Fish meal

## Discussion

A plethora of studies have been conducted to replace the costly and scarcely available FM with other protein sources in aquaculture. Rice protein concentrate is considered the most feasible PP source and is used as a substitute for expensive FM, has a high nutrient content and is easily available. Nonetheless, the presence of ANF, such as phytate, hinders its utilization in fish [31, 32]. To maintain aquaculture sustainability, it is imperative to address and find solutions to this problem. Therefore, the current study aimed to overcome this deficiency by supplementing an RPC-based diet with phytase, an exogenous enzyme. The *in vitro* hydrolysis of phytate P in the RPC-based diet increased as the phytase supplementation level increased in the present study up to 1000 FTU/kg, which is in accordance with the findings reported by Baruah et al. [29] and Shah et al. [33]. This suggests that increasing the phytase levels in RPC-based diets increases P release, which might enhance P availability in fish [33]. However, there may be a maximum inclusion level of phytase, and the acceptability of this substitution concerning fish growth performance has exhibited considerable variation among various fish species [34–36].

The present study demonstrated that dietary supplementation with phytase supplemented with RPC, a substitute for FM, had positive effects on growth performance up to 1000 FTU/kg. Similar findings have been reported for Nile tilapia [37–39], African catfish [8], channel catfish [40], rohu [41], and Pacific white shrimp [42]. Phytase decreases phytate contents in diets, which might release bound nutrients, especially P, and improve the digestion of protein-bound phytate. Thus, phytase supplementation improved protein utilization and overall nutritional efficiency in fish [19, 43–45]. Nevertheless, supplementation above the required level reduced the utilization of feed and growth performance, possibly due to the degradation of nutrients present in the feed, e.g., proteins and amino acids, from the overdose of phytase [18, 38, 39, 40]. Therefore, the broken line regression analysis of the weight gain % revealed that the recommended level of phytase supplementation for *L*. *rohita* fingerlings is estimated to be 874.19 FTU/kg. The HSI and VSI are indicators used to evaluate the effects of environmental or dietary manipulations on fish. In the present study, no changes in the HSI or VSI were observed, which indicates that there was no nutritional deficiency, disease, or environmental stressor in the fish; these findings are consistent with previous reports on various fish species [11, 24].

The carcass composition, such as moisture content, CP, CF, and ash content, is a reliable parameter for estimating the nutrient profile of fish and is crucial for consumers [46]. Phytate complex formation is reported to hinder the availability of nutrients such as CP and CF to fish species, preventing their utilization [47, 48]. No alteration in body composition in the present study indicated no negative effect on the nutrient profile, which is consistent with the findings of previous reports examining gibel carp and tilapia [49, 50] and might be due to the hydrolysis of phytate P. The P release from phytate enhances the utilization of P at the highest supplementation level of a PP-based diet [51]. In contrast to our study, a significant alteration was observed, which might be due to the presence of NADPH in the PP sources, which ultimately affects the lipogenic pathway and the production of ATP [42, 48, 52].

Minerals are vital for facilitating the growth, development, and overall well-being of fish and play vital roles in various physiological processes. Phytate is a strong chelator that can bind to minerals (Fe, Ca, and Mg), forming complexes that are insoluble and unavailable for absorption by fish [17]. The present study revealed that *L*. *rohita* fingerlings supplemented with phytase had higher mineral contents (P, Ca, Mg, Na, K, Mn, Cu, and Zn) in the whole body and bones, up to 1000 FTU/kg phytase, than did the fish fed diets with no phytase enzyme, which could be attributed to the efficacy of the enzyme in hydrolyzing the phytate content, leading to the liberation of bound minerals that were subsequently utilized and retained by the fish [48]. Similarly, Olugbenga et al. [53] reported that the addition of phytase at 750–1000 FTU/kg enhanced the mineral content of African catfish. Similar results were observed in various fish species, e.g., crucian carp, African catfish, and grass carp [53–56].

The digestibility of alternative protein sources as ingredients plays a crucial role in assessing their suitability in the formulation of fish feed [57]. Phytate binds with other enzymes and components present in the diet, ultimately lowering nutrient digestibility and activating specific enzymes as well as potentially affecting culture conditions [16, 43, 58, 59]. The present study revealed that 1000 FTU/kg phytase supplementation significantly improved the ADC, which is consistent with the findings of previous studies [58, 60]. However, no effect on the ADC of nutrients was shown for some fish species when dietary phytase was added to their feeds [61]. In the present study, the increase in phytase supplementation in an RPC-based diet increased the mineral digestibility of Ca, Mg, P, Na, K, Zn, Mn, and Cu. Similarly, previous findings have indicated that phytase addition enhances mineral absorption in many fish species under different cultures and environmental conditions [54, 59, 62–65]. The observed phenomenon can be attributed to phytate hydrolysis facilitated by phytase supplementation, as is evident in *in vitro* phytate hydrolysis. In contrast to the current study, the tiger puffer *Takifu gurubripes* [66] showed greater mineral digestibility at the 2000 FTU/kg phytase level. Moreover, Nwanna and Olusola [67] suggested that phytase addition to PP-based diets had no effect on the ADC of minerals in *Oreochromis niloticus*.

Amino acid transaminases, namely, AST and ALT, are integral components of metabolic processes and are found primarily within hepatocytes. In aquatic animals, the activities of these enzymes in serum may serve as markers of the overall health of the liver [68, 69]. The increased concentrations of ALT and AST are discharged into the serum, which may indicate damage to liver cells [70]. ALP is crucial for membrane transport and mineralization of the skeleton in aquatic species [11]. In the present study, the activities of ALT, AST, and ALP decreased with phytase supplementation in response to RPC-based diets, which was inconsistent with the findings of previous studies by Liu et al. [71] on *Ctenopharyngdon idella* and Yang et al. [11] on red swamp crayfish (*Procambarus clarkia)*. In contrast, no effect was observed for different fish species when phytase was supplemented in PP-based diets [36, 37, 72].

Digestive enzymes such as protease, lipase, and amylase mostly represent the ability to digest and absorb nutrients, which helps in reproduction and enhanced growth performance in many fish species [73, 74]. Phytate binds to digestive enzymes or their cofactors and affects their efficacy [75]. The current study demonstrated that dietary phytase addition increased the intestinal digestive enzyme activity of *L*. *rohita*. The addition of phytase dissolved the phytate by breaking down the complexes and discharging the bound minerals, hence playing a crucial role in enhancing enzyme activity [11]. Similar results have been reported in other fish species, e.g., rohu, red swamp crayfish, and Nile tilapia [11, 70, 76].

## Conclusion

In conclusion, RPC in combination with phytase is a viable and efficient substitute protein source for promoting sustainable aquaculture practices and maintaining the health and productivity of *L*. *rohita* without causing any significant negative effects on growth performance, digestibility, mineral status, digestive enzymes, or serum biochemistry. The optimum fish performance was observed with the inclusion of 1000 FTU/kg phytase containing 32% RPC. However, the phytase level in the diet of *L*. *rohita* fingerlings determined via broken line regression analysis of weight gain (%) data was calculated to be 874.19 FTU/kg when 6.8 g/kg phytate was present in the RPC-based diet.

## Supporting information

S1 Raw data This is the link of S1 Raw data. https://figshare.com/s/63b4fea61c3438a187c3, **(doi**: 10.6084/m9.figshare.25688556).
(XLSX)
